# An Optimized Protocol for the Generation of Alveolospheres from Wild-Type Mice

**DOI:** 10.3390/cells13110922

**Published:** 2024-05-27

**Authors:** Mahsa Zabihi, Ali Khadim, Theresa M. Schäfer, Ioannis Alexopoulos, Marek Bartkuhn, Elie El Agha, Ana I. Vazquez-Armendariz, Susanne Herold

**Affiliations:** 1 Department of Medicine V, Internal Medicine, Infectious Diseases and Infection Control, Universities of Giessen and Marburg Lung Center (UGMLC), German Center for Lung Research (DZL), Justus-Liebig University Giessen (JLU), 35392 Giessen, Germany; mahsa.zabihi@innere.med.uni-giessen.de (M.Z.); ali.khadim@innere.med.uni-giessen.de (A.K.); theresa.m.schaefer@innere.med.uni-giessen.de (T.M.S.); ioannis.alexopoulos@innere.med.uni-giessen.de (I.A.); 2 Cardio-Pulmonary Institute (CPI), 35392 Giessen, Germany; 3 Institute for Lung Health (ILH), 35392 Giessen, Germany; 4 Biomedical Informatics and Systems Medicine, Justus-Liebig University Giessen (JLU), 35392 Giessen, Germany; marek.bartkuhn@gen.bio.uni-giessen.de; 5 Transdisciplinary Research Area Life and Health, Organoid Biology, Life & Medical Sciences Institute, University of Bonn, 53115 Bonn, Germany

**Keywords:** lung organoids, epithelial stem and progenitor cells, mesenchymal cells, type 2 alveolar epithelial cells (AEC2s), alveolospheres

## Abstract

Organoid models have become an integral part of the research methodology in the lung field. These systems allow for the study of progenitor and stem cell self-renewal, self-organization, and differentiation. Distinct models of lung organoids mimicking various anatomical regions of mature lungs have emerged in parallel to the increased gain of knowledge regarding epithelial stem and progenitor cell populations and the corresponding mesenchymal cells that populate the in vivo niche. In the distal lung, type 2 alveolar epithelial cells (AEC2s) represent a stem cell population that is engaged in regenerative mechanisms in response to various insults. These cells self-renew and give rise to AEC1s that carry out gas exchange. Multiple experimental protocols allowing the generation of alveolar organoids, or alveolospheres, from murine lungs have been described. Among the drawbacks have been the requirement of transgenic mice allowing the isolation of AEC2s with high viability and purity, and the occasional emergence of bronchiolar and bronchioalveolar organoids. Here, we provide a refined gating strategy and an optimized protocol for the generation of alveolospheres from wild-type mice. Our approach not only overcomes the need for transgenic mice to generate such organoids, but also yields a pure culture of alveolospheres that is devoid of bronchiolar and bronchioalveolar organoids. Our protocol contributes to the standardization of this important research tool.

## 1. Introduction

Organoids are three-dimensional (3D) structures that are representative of the structure, cellular morphology, and certain functional features of the tissue or organ of interest. In recent years, murine and human organoids have emerged as powerful tools for studying organ development, cellular interaction, drug screening, and regenerative mechanisms. Due to their unlimited capacity to proliferate and differentiate, adult stem and progenitor cells have been widely used to develop organoids for research purposes [1,2].

In the context of pulmonary research, several murine organoid systems have been developed to create airway and/or alveolar structures resembling various aspects of lung organogenesis, homeostasis, and regeneration [3]. For instance, tracheal organoids, or tracheospheres, with a visible lumen can be grown from basal stem cells even in the absence of stromal cells or other non-basal cells [4]. Airway organoids, or bronchiolospheres, can be generated by co-culturing club cells with airway smooth muscle cells (ASMCs) or repair-supportive mesenchymal cells (RSMCs), thus mimicking the intercellular communication that occurs during airway epithelial regeneration in vivo following naphthalene injury [5,6,7,8]. Such bronchiolospheres can also be generated by culturing club cells with [CD45− CD31− EpCAM− Sca-1+] (non-leukocytic, non-endothelial, non-epithelial, stem cell antigen-1-expressing) resident mesenchymal cells (rMCs) [5]. The most comprehensive system so far is the bronchioalveolar lung organoid (BALO) model, which relies on co-culturing bronchioalveolar stem cells (BASCs) and rMCs, resulting in a highly sophisticated mimicry of the bronchioalveolar region of the mature mouse lung [7,9].

Type 2 alveolar epithelial cells (AEC2s) represent a stem cell population in the alveolar compartment of the lung [10]. These cells self-renew and regenerate this compartment by differentiating into type 1 alveolar epithelial cells (AEC1s) that are responsible for gas exchange, eventually via intermediate cell types [11,12,13]. Although the heterogeneous nature of AEC2s was not obvious initially, emerging research suggests that there are indeed AEC2 subsets that display varying stem-cell features. For example, WNT-responsive AEC2s (expressing axis inhibition protein 2 or AXIN2), termed alveolar epithelial progenitors (AEPs), have been shown to represent a stable lineage during homeostasis, and are capable of rapid expansion following acute lung injury [14]. Another subpopulation of AEC2s is enriched in programmed cell death 1 ligand 1 (PD-L1 or CD274) expression and was dubbed “injury-associated alveolar progenitors” (IAAPs). These cells replenish the mature AEC2 pool upon alveolar injury [15,16].

Isolation of AEC2s by FACS for alveolosphere cultures has so far mostly relied on the use of genetically modified mice (*Sftpc^Cre-ERT2/+^*; *tdTomato^flox^*) that allow labeling of surfactant protein C-positive (SFTPC+) AEC2s. Such cells can also be filtered through the additional use of Lysotracker, a dye that stains lysosomes and therefore detects lamellar bodies that are abundant in AEC2s [17]. A population of rMCs that expresses fibroblast growth factor 10 (FGF10) and contains neutral lipids (therefore possessing a lipofibroblast-like phenotype) promotes the formation of AEC2-derived alveolospheres containing AEC2s and AEC1s [18,19]. Alveolospheres had been initially described by co-culturing lineage-labeled AEC2s with platelet-derived growth factor receptor alpha (PDGFRα)-expressing mesenchymal cells, also termed type 2-associated stromal cells (TASCs), that display lipofibroblast characteristics [10,20]. The co-culture assay using Sca-1+ mesenchymal cells and lung epithelial progenitor cells had already been developed [21].

To overcome the need for transgenic mice for the generation of alveolospheres, we hereby provide a detailed protocol for culturing such organoids starting with wild-type (WT) mouse lungs. We validated various aspects of our approach including colony-forming efficiency (CFE), alveolosphere diameter, and cellular differentiation by comparison with alveolospheres obtained from *Sftpc^Cre-ERT2/+^*; *tdTomato^flox^* mice. Our protocol serves as a resource for the lung stem-cell research community interested in AEC2 self-renewal and differentiation, and interaction with mesenchymal niche cells.

## 2. Materials and Methods

### 2.1. Mice and Tamoxifen Administration

Mice between 8 and 12 weeks of age were used and maintained on the C57BL/6 background. Male wild-type (WT) mice were purchased from Charles River (Strain code 632, Wilmington, MA, USA). Sftpc^tm1(Cre/ERT2,rtTA)Hap^ (or simply *Sftpc^Cre-ERT2^*) mice were previously described [22], and Gt(ROSA)26Sor^tm9(CAG-tdTomato)Hze^ (or simply *tdTomato^flox^*) mice were purchased from the Jackson Laboratory (stock number 007905, Bar Harbor, ME, USA). The two lines were bred to generate *Sftpc^Cre-ERT2/+^*; *tdTomato^flox^* mice. Tamoxifen powder (Sigma-Aldrich, T5648, St. Louis, MO, USA) was dissolved in corn oil (Sigma-Aldrich, C8267) and injected intraperitoneally three times at a dose of 0.25 mg/g body weight per injection. Mice were kept under specific pathogen-free (SPF) conditions with unlimited access to food and water.

### 2.2. Lung Dissociation and Fluorescence-Activated Cell Sorting

Lungs from C57BL/6 (WT) and *Sftpc^Cre-ERT2/+^*; *tdTomato^flox^* mice were collected for single-cell suspension preparation, as previously described [16]. Briefly, the lungs were flushed via transcardiac perfusion using 20 mL of Hank’s balanced salt solution (HBSS). Digestion was performed by intratracheal injection of 2 mL of dispase (5 U/mL; BD Biosciences, 354235, Franklin Lakes, NJ, USA) and incubation in 3 mL of dispase solution (5 U/mL) at room temperature (RT) for 40 min. After digestion, lung homogenates were passed through 70 µm and 40 µm cell strainers. The cell suspension was centrifuged and incubated with an antibody cocktail containing biotin rat anti-mouse CD45 (BD Biosciences, 553078), CD16/32 (BD Biosciences, 553143), and CD31 (BD Biosciences, 553371) at 37 °C for 30 min to deplete hematopoietic and endothelial cells. After centrifugation, cells were resuspended in DMEM (Dulbecco’s Modified Eagle Medium) containing Dynabeads Biotin Binder (Invitrogen, 11047, Waltham, MA, USA) for 30 min at RT on a rotator. For magnetic separation, cells were transferred to a magnetic rack for 10 min at RT. Cell suspensions were then blocked with gamma globulins (Gamunex 10%; Grifols, Barcelona, Spain), re-suspended in MASC buffer (PBS 1X, FBS 0.5%, UltraPure EDTA (Invitrogen, 15575-038, 2 mM)) and stained with anti-mouse CD31 (Alexa Fluor 488, Biolegend, 102514, 1:50, San Diego, CA, USA), CD45 (FITC, BD Biosciences, 553080, 1:50), CD326 (APC/Cy7, Biolegend, 118218, 1:50), Sca-1 (Pacific Blue, Biolegend, 108120, 1:50, Santa Monica, CA, USA), and Podoplanin antibodies (APC, Biolegend, 127410, 1:50) for 15 min at 4 °C. Next, CD45− CD31− EpCAM− Sca-1+ rMCs and CD45− CD31− EpCAM^low^ AECs were isolated by FACS. At least 40,000 rMCs and 350,000 AECs from one WT mouse and 250,000 tdTomato+ (tdTom+) cells from one TG mouse can be sorted. To get a higher yield of rMCs, WT lungs can be separately digested with collagenase type IV as previously described [6]. Sorting was performed using FACSAria III cell sorter (BD Biosciences). Data were analyzed using FlowJo software, version 10.10.0 (FlowJo LLC, Ashland, OR, USA).

### 2.3. Alveolosphere Generation and Analysis

To generate alveolospheres, 17,000 rMCs and 5000 AECs from WT and *Sftpc^Cre-ERT2/+^*; *tdTomato^flox^* mice were flow-sorted, mixed, and resuspended in 50 µL of culture medium containing minimum essential medium (MEM) alpha (Gibco, 41061029, Waltham, MA, USA), 10% Fetal Bovine Serum (FBS) (Gibco, 10270-106), 1% L-glutamine–penicillin–streptomycin (Sigma-Aldrich, G1146), 1% insulin/transferrin/selenium (ITS) (Gibco, 41400-045), and 0.0002% heparin (Stem Cell Technologies, 07980, Waltham, MA, USA). Next, 50 µL of cold growth factor-reduced Phenol Red-free Matrigel (Corning, 356231, Corning, NY, USA) was mixed at a 1:1 ratio with the cell suspension, transferred to a 24-well plate on a 0.4 µm cell culture insert (Millipore, PICM01250, Burlington, MA, USA), and incubated at 37 °C for 15 min for polymerization. After incubation, 350 µL of medium was added to the lower chamber and incubated at 37 °C with 5% CO_2_ for up to 16 days. To test the effect of IL-1β on alveolosphere formation, recombinant murine IL-1β (PeproTech, 211-11B-10UG, Waltham, MA, USA) was added to the organoid medium at a concentration of 20 ng/mL. The medium was changed every 2 days. Cultures were imaged using EVOS M7000 (Thermo Fisher, AMF7000, Waltham, MA, USA). The number and area of alveolospheres were measured by ImageJ (v2.14.0/1.54f). The colony-forming efficiency (CFE) was determined by dividing the number of counted alveolospheres by the initial number of seeded AECs.

### 2.4. Induction of Cre Activity in Ex Vivo Alveolosphere Cultures

To induce Cre-mediated recombination ex vivo, organoid medium containing 1 µM of 4-Hydroxytamoxifen (4-OHT) (Merck, H6278, Darmstadt, Germany) was added to the organoid cultures on day 1. After 24 h, the organoid medium containing 4-OHT was removed, and the organoid inserts were washed twice with 1X PBS. Then, the inserts were placed on a new 24-well plate and alveolospheres were cultured in organoid medium until day 16.

### 2.5. Whole-Mount Immunofluorescence

On day 16 of culture, alveolospheres were fixed with 4% paraformaldehyde (PFA) (Merck, 104005) for 20 min at RT, then washed three times with 1X PBS. Fixed alveolospheres were blocked and permeabilized with blocking buffer (5% serum, 0.5% Triton-X-100 in 1X PBS) overnight at 4 °C. The serum was chosen from the same species as the host of the secondary antibody. For immunofluorescence, alveolospheres were incubated with primary antibodies in a blocking buffer overnight at 4 °C. The next day, washing buffer (2% serum, 0.3% Triton-X-100 in 1X PBS) was added 3 times for 10–15 min, and secondary antibodies were added overnight at 4 °C. To stain nuclei, the NucBlue™ Fixed Cell ReadyProbes™ Reagent (Thermo Fisher) was utilized. Lastly, stained alveolospheres were washed three times with washing buffer for 10–15 min. The Matrigel containing the organoids was cut out of the insert with a scalpel. The sample was placed on a slide and covered with 5–6 drops of fructose–glycerol clearing solution (60% glycerol, 2.5 M fructose) [23]. A coverslip was placed on the sample and after drying for 1 day at 4 °C in the dark, imaging was carried out using an SP8 confocal microscope (Leica Microsystems, Wetzlar, Germany) controlled by LAS X software (version 3.5.7). For capturing the confocal z-stacks, we used 20x0.75NA air and 63xW 1.2NA objectives, while the scan settings were kept the same across all acquisitions. A list of primary and secondary antibodies can be found in Table 1.

### 2.6. Image Analysis

The acquired confocal images were cleared computationally, using the Lightning module of LAS X software. Subsequently, for the maximum intensity projection and quantification of the z-stack, we used the ImageJ/Fiji software [24]. With Fiji, every projected image was thresholded using the default integrated algorithm. Then, the area occupied by the positive signal (of each marker) as well as the total organoid area were measured. The final area ratio of each marker was calculated by dividing the marker’s area by the organoid’s area.

### 2.7. RNA Extraction and Gene Expression Analysis

RNA was extracted from alveolosphere cultures using the RNeasy micro kit (Qiagen, 74004, Hilden, Germany). cDNA synthesis was performed using the Quantitect reverse transcription kit (Qiagen, 205311) according to the manufacturer’s instructions. Quantitative real-time PCR (qPCR) was performed using a PowerUp SYBR green master mix (Thermo Fisher Scientific, A25742) and a LightCycler 480 II machine (Roche Applied Science, Penzberg, Germany). Gene expression levels were normalized to glyceraldehyde-3-phosphate dehydrogenase (*Gapdh*) and presented as fold change relative to day 8 of culture. Primer sequences are shown in Table 2. Bulk RNA sequencing was carried out according to standard procedures. For genome-wide analysis of gene expression, RNA sequencing libraries from polyadenylated mRNA were generated and sequenced using Illumina NovaSeq 6000 at the Institute for Lung Health (ILH)-Genomics and Bioinformatics at the Justus-Liebig University (JLU) Giessen (Germany).

### 2.8. Statistical Analysis

The number of biological replicates is provided in the corresponding figure legends. All data are presented as mean ± standard error of the mean (SEM). Student’s *t* test (unpaired, two tailed) was used to compare the means of two groups and One-way ANOVA was used to compare more than two groups. The differences in the means were considered statistically significant when the *p* value was less than 0.05. Data were assembled and analyzed using GraphPad Prism software, version 9.5.1 (528) (GraphPad Software, Boston, MA, USA).

## 3. Results

### 3.1. AEC2s Can Be Isolated from WT Mouse Lungs by Sorting CD45− CD31− EpCAM^low^ Cells

**Figure 1:**
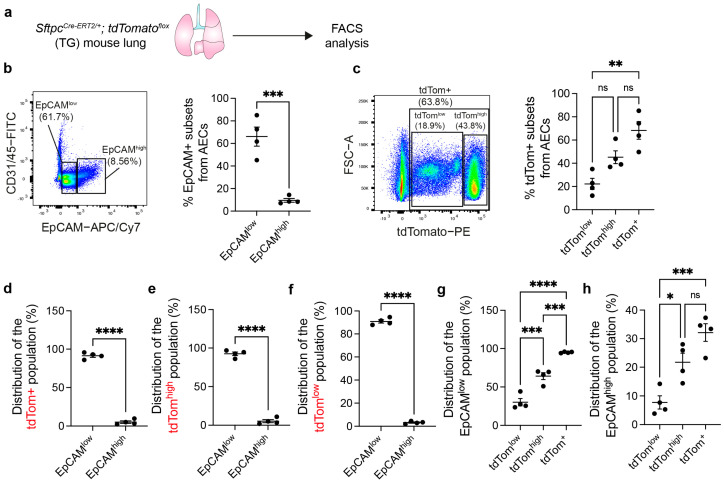
Gating strategy to identify AEC2s within the EpCAM^low^ cell population. (**a**) Experimental design for FACS analysis from TG mouse lungs. (**b**,**c**) Gating strategy for identifying EpCAM^low^, EpCAM^high^, tdTom^low^, and tdTom^high^ populations with the corresponding quantification. (**d**–**f**) Distribution of tdTom+, tdTom^high^, and tdTom^low^ cells among EpCAM^low^ and EpCAM^high^ populations. (**g**,**h**) Distribution of EpCAM^low^ and EpCAM^high^ cells among tdTom^low^, tdTom^high^, and tdTom+ populations. Each dot represents one lung; * *p* < 0.05; ** *p* < 0.01; *** *p* < 0.001; **** *p* < 0.0001; ns: not significant. AECs: alveolar epithelial cells; TG: transgenic.

To show a proof of concept that alveolospheres can be generated from AECs that are FACS sorted from WT lungs, we first carried out EpCAM staining on AEC cell suspensions prepared from *Sftpc^Cre-ERT2/+^; tdTomato^flox^* lungs (Figure 1a). We identified EpCAM^low^ (66.1% ± 8.4% of AECs) and EpCAM^high^ (9.6% ± 1.6% of AECs) populations (Figure 1b). We have previously demonstrated that the EpCAM^low^ population is enriched with AEC2s [19], while the EpCAM^high^ cell population is enriched with airway cells such as ciliated and secretory cells [19] as well as bronchioalveolar stem cells (BASCs) [9]. Moreover, tdTom^low^ (22.2% ± 4.8% of AECs) and tdTom^high^ (45.2% ± 5.5% of AECs) corresponding to injury-activated alveolar progenitors (IAAPs) and mature AEC2s, respectively, were also identified (Figure 1c) as previously described [16]. IAAPs have been shown to be inefficient in generating alveolospheres [16]. Total tdTom+ cells accounted for around two-thirds of the AEC preparation (Figure 1c), similar to EpCAM^low^ cells (Figure 1b).

Gating for total tdTom+ cells (tdTom^low^ and tdTom^high^) showed that 91.4% ± 2.1% of these cells were contained in the EpCAM^low^ population compared to traces in EpCAM^high^ cells (Figure 1d). Gating for tdTom^high^ and tdTom^low^ populations showed similar results (Figure 1e,f). Further analysis of the EpCAM^low^ population showed that 95% ± 0.42% of these cells were tdTom+, with the majority being tdTom^high^ (64.1% ± 4.5%) and a minor proportion of tdTom^low^ (30.2% ± 4.7%) (Figure 1g). On the other hand, the EpCAM^high^ population showed lower proportions of tdTom+ cells (Figure 1h). Therefore, the EpCAM^low^ population was confirmed to be enriched with AEC2s.

**Figure 2:**
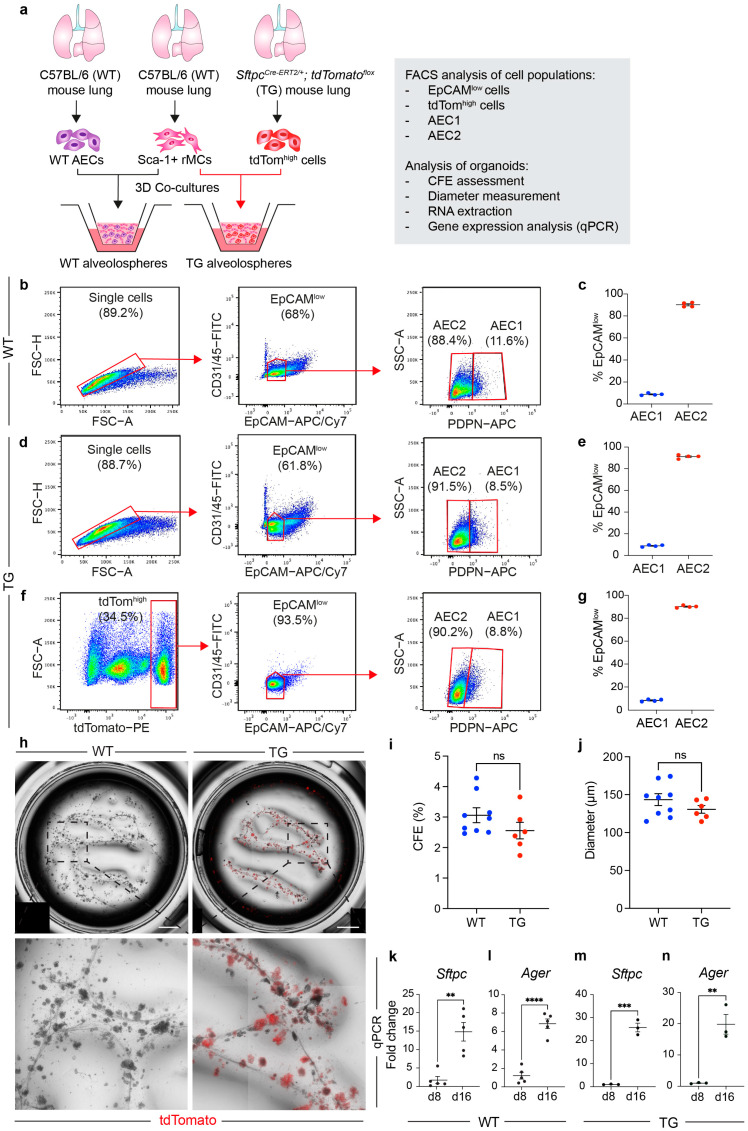
Generation of alveolospheres from WT and TG mouse lungs. (**a**) Experimental design for co-culturing WT or TG AEC2s with rMCs and subsequent analysis. (**b**) Gating strategy for sorting AEC2s (EpCAM^low^) from WT mouse lungs. (**c**) Quantification of AEC1s and AEC2s in the EpCAM^low^ population. (**d**) Gating strategy for identifying AEC2s (EpCAM^low^) in TG mouse lungs. (**e**) Quantification of AEC1s and AEC2s in the EpCAM^low^ population. (**f**) Gating strategy to select EpCAM^low^ cells from the tdTom^high^ population. (**g**) Quantification of AEC1s and AEC2s in the EpCAM^low^ fraction of the tdTom^high^ population. (**h**) Tile scans of representative wells for WT and TG alveolosphere cultures. (**i**,**j**) Quantification of CFE and alveolosphere diameter. (**k**–**n**) qPCR for alveolar epithelial marker genes on days 8 and 16 of culture. (**c**,**e**,**g**) Each dot represents one lung; (**i**,**j**) each dot represents one culture well; (**k**–**n**) each dot represents two pooled culture wells. ** *p* < 0.01; *** *p* < 0.001; **** *p* < 0.0001; ns: not significant. AECs: alveolar epithelial cells; CFE: colony-forming efficiency; rMCs: resident mesenchymal cells; TG: transgenic; WT: wild type. Scale bars: 1 mm.

Next, rMCs were co-cultured with either AECs isolated from WT lungs (hereafter referred to as the WT approach) or tdTom+ cells from *Sftpc^Cre-ERT2/+^; tdTomato^flox^* lungs (hereafter referred to as the transgenic (TG) approach) (Figure 2a). WT AECs were sorted from single-cell suspensions based on CD45− CD31− EpCAM^low^ surface marker expression (Figure 2b). The majority of isolated cells were identified as CD45− CD31− EpCAM^low^ PDPN− AEC2s (90.2% ± 1.01% from the parent EpCAM^low^ gate), while only 8.8% ± 0.47% was CD45− CD31− EpCAM^low^ PDPN+ AEC1s (Figure 2c). Lungs from *Sftpc^Cre-ERT2/+^; tdTomato^flox^* mice were similarly processed for FACS sorting. Staining for EpCAM and PDPN revealed comparable proportions of AEC2s and AEC1s within the EpCAM^low^ population similar to the WT approach (Figure 2d,e). Since IAAPs are quiescent in the absence of injury and therefore do not robustly generate alveolospheres [16], tdTom^high^ cells were initially selected for alveolosphere generation (Figure 2f). As shown in Figure 1e, 92.5% ± 2.4% of tdTom^high^ cells are found in the EpCAM^low^ gate. Staining for PDPN revealed that 90.1% ± 0.5% of tdTom^high^ EpCAM^low^ cells were AEC2s, while only 8.46% ± 0.45% were AEC1s (Figure 2g), resembling the data attained with WT EpCAM^low^ cells.

### 3.2. The EpCAM^low^ Population Robustly Generates Alveolospheres and It Is Comparable with Sorted Lineage-Traced AEC2s

The data described above clearly show that gating for CD45− CD31− EpCAM^low^ cells in WT lungs allows for the sorting of an AEC population that predominantly consists of AEC2s. The finding that lineage-traced AEC2s essentially belong to the EpCAM^low^ population (Figure 1d–f) strongly supports this conclusion. We therefore tested the ability of EpCAM^low^ cells to generate alveolospheres (WT approach) and compared the results to the TG approach. As expected, all alveolospheres in the TG approach cells were tdTom+ (Figure 2h). Both approaches were similarly efficient in generating alveolospheres (WT CFE 3.14% ± 0.23% vs. TG CFE 2.55% ± 0.27%) (Figure 2i) with comparable alveolosphere diameter (139.37 μm ± 6.86 μm for WT vs. 130.57 μm ± 5.01 μm for TG) (Figure 2j). Gene expression analysis was then carried out on WT and TG alveolospheres on day 8 (when AECs start to form cysts) and day 16 (when alveolospheres acquire a complex morphology and contain differentiated AEC2s and AEC1s). The results showed significant upregulation of the AEC2 marker *Sftpc* (Figure 2k,m) and the AEC1 marker *Ager* (Figure 2l,n) in both conditions.

### 3.3. WT Alveolospheres Derive from Isolated AEC2s

**Figure 3:**
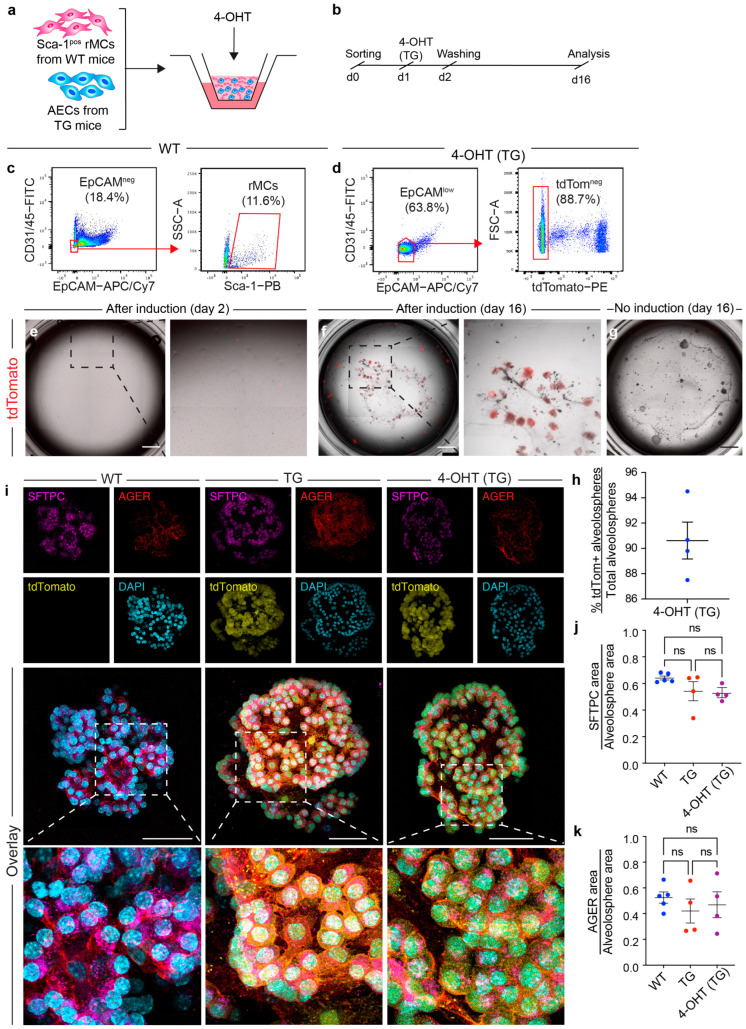
Alveolospheres derive from SFTPC+ AEC2s and contain both AEC1s and AEC2s. (**a**,**b**) Experimental design for co-culturing uninduced TG AEC2s with WT rMCs and ex vivo induction with 4-OHT. (**c**,**d**) Gating strategy for sorting WT rMCs and tdTom− EpCAM^low^ cells from uninduced TG mouse lungs. (**e**,**f**) Tile scans of representative wells on days 2 and 16. The dashed boxes are magnified in the right panels. The tdTom signal is shown in red. (**g**) Tile scan of a day 16 culture well in the absence of 4-OHT. (**h**) Quantification of the percentage of tdTom+ alveolospheres. (**i**) Representative images of the maximum intensity projection images of individual organoids from the indicated conditions stained for SFTPC and AGER. Note the presence of the tdTom+ signal in the TG and 4-OHT (TG) conditions but not the WT condition. (**j**,**k**) Quantification of the ratio of the SFTPC and AGER signals to alveolosphere area. (**h**) Each dot represents one culture well; (**j**,**k**) each dot represents one organoid. 4-OHT: 4-hydroxytamoxifen; AECs: alveolar epithelial cells; ns: not significant; rMCs: resident mesenchymal cells; TG: transgenic; WT: wild type. Scale bars: (**e**–**g**) 1 mm; (**i**) 50 μm.

To validate that the organoids indeed derive from AEC2s, the CD45− CD31− EpCAM^low^ tdTom− AEC population was isolated from uninduced *Sftpc^Cre-ERT2/+^; tdTomato^flox^* lungs and co-cultured with WT rMCs. Cre-mediated recombination was induced ex vivo by exposure to the active metabolite of tamoxifen, 4-OHT, between days 1 and 2 (Figure 3a–d). By day 2, cultures revealed single tdTom+ cells (Figure 3e), with most alveolospheres (90.61% ± 1.45%) being tdTom+ on day 16 (Figure 3f,h). In the absence of 4-OHT, organoids were negative for tdTom (Figure 3g). These data strengthen the notion that the EpCAM^low^ population indeed enriches for AEC2s that are capable of ex vivo expansion and subsequent alveolosphere formation.

Alveolospheres generated using the WT, TG, and 4-OHT (TG) approaches were then subjected to immunofluorescence for SFTPC and AGER, followed by whole-mount confocal imaging (Figure 3i). The data revealed similar ratios relative to alveolosphere area among the three groups for SFTPC (WT 0.64 ± 0.01 vs. TG 0.54 ± 0.07 vs. 4-OHT (TG) 0.52 ± 0.02) (Figure 3j) and AGER (WT 0.52 ± 0.04 vs. TG 0.42 ± 0.09 vs. 4-OHT (TG) 0.46 ± 0.1) (Figure 3k), without apparent differences in terms of overall morphology, suggesting that the cellular composition within these conditions is highly comparable.

**Figure 4:**
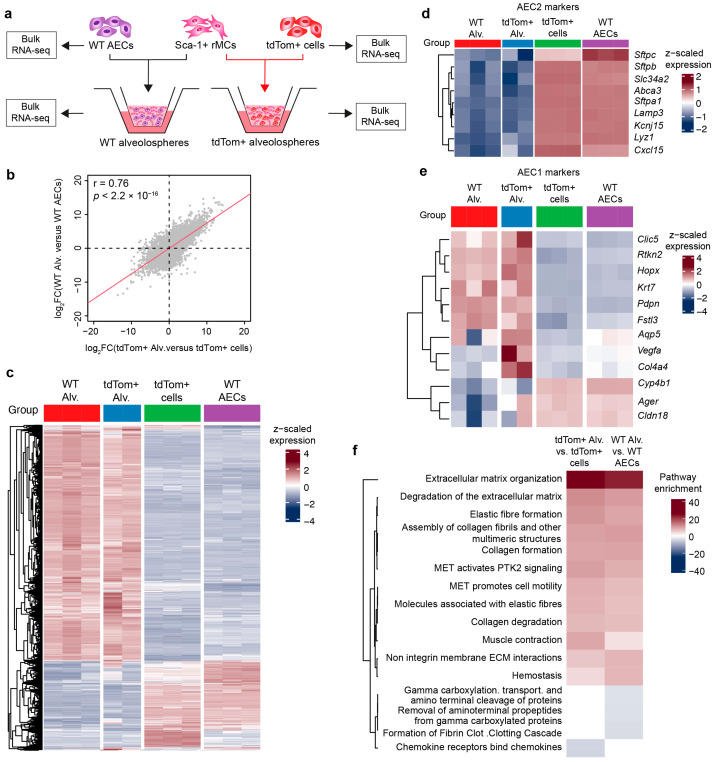
Resemblance between the WT and TG approaches at the transcriptomic level. (**a**) Experimental design. (**b**) Scatter plot showing the correlation between the WT and TG approaches. (**c**) Heatmap showing the top 2000 differentially expressed genes between alveolospheres and sorted cells. (**d**,**e**) Heatmaps showing selected AEC2 and AEC1 marker genes in various samples. (**f**) Reactome pathway analysis of the top differentially expressed genes in alveolospheres versus sorted cells. Pathway enrichment was calculated by multiplying −log_10_(*p* value) by +1 (in case of upregulation) or −1 (in case of downregulation). Alv.: alveolospheres; rMCs: resident mesenchymal cells; TG: transgenic; WT: wild type.

To further demonstrate that the alveolospheres generated using the WT approach are comparable to those generated using the TG approach, we carried out RNA-seq on isolated WT AECs, tdTom+ cells, and the corresponding organoids (Figure 4a). The full list of genes is shown in Supplementary Table S1. Notably, the same gene sets are modulated during organoid formation regardless of whether the starting material is tdTom+ cells or WT AECs (Figure 4b,c). Moreover, a comparison of AEC2 and AEC1 markers showed no difference between the two groups (Figure 4d,e), confirming that the cell population isolated using our WT EpCAM^low^ approach is very similar to the tdTom+ population isolated from transgenic animals. We also found that the AEC1 signature is upregulated in alveolospheres compared with isolated AEC2s, while the AEC2 signature is diluted in alveolospheres (Figure 4d,e), which is expected given the emergence of AEC1s and the abundance of mesenchymal cells in the alveolospheres but not in purified AEC2s. Pathway analysis of the top genes differentially regulated between alveolospheres and AEC2s was related to ECM organization, elastic fibers, collagen biosynthesis, and cell motility (Figure 4f).

### 3.4. Stimulation with Recombinant Murine IL-1β Alters Alveolosphere Formation

**Figure 5:**
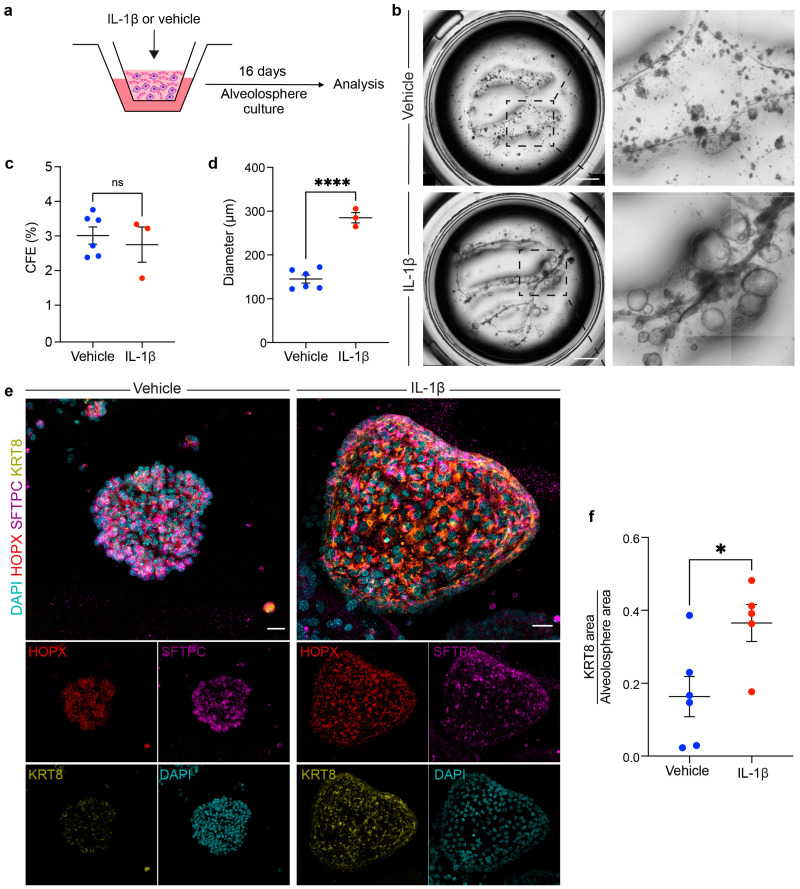
Treatment with recombinant IL-1β impacts alveolosphere morphology and differentiation. (**a**) Experimental design for co-culturing WT AEC2s with WT rMCs in the presence or absence of IL-1β. (**b**) Tile scans of representative wells on day 16. The dashed boxes are magnified in the right panels. (**c**,**d**) Quantification of CFE and alveolosphere diameter. (**e**) Representative maximum intensity projection images of individual organoids from the indicated conditions stained for HOPX, SFTPC, and KRT8. (**f**) Quantification of the ratio of the KRT8 signal to alveolosphere area. (**c**,**d**) Each dot represents one culture well; (**f**) each dot represents one organoid. * *p* < 0.05; **** *p* < 0.0001; ns: not significant. CFE: colony-forming efficiency. Scale bars: (**b**) 1 mm; (**e**) 50 μm.

It has been reported that IL-1β treatment leads to enlarged alveolospheres that feature accumulation of the KRT8+ intermediate cell state [12]; therefore, recombinant IL-1β was added to WT cultures as another proof of concept that such organoids behave similarly to those obtained using the TG approach (Figure 5a). After 16 days of culture, organoids clearly showed a cyst-like morphology rather than a typical alveolosphere morphology in the IL-1β group (Figure 5b), which was also reflected by the quantification of organoid diameter (285.08 μm ± 11.6 μm vs. 145.04 μm ± 9.16 μm) (Figure 5d). Analysis of the CFE did not reveal a difference between the two groups (Figure 5c). Notably, immunofluorescence showed a significant increase in the coverage of the KRT8 signal relative to the overall alveolosphere area upon IL-1β treatment (0.36 ± 0.05 vs. 0.16 ± 0.05) (Figure 5e,f). These data indicate that our WT approach can recapitulate previous findings attained using the TG approach.

## 4. Discussion

Over the past decade, lung organoids have emerged as important models to investigate morphogenic and regenerative aspects of lung biology and disease such as assessing epithelial stem/progenitor cell self-renewal, self-organization, and differentiation, as well as the supportive potential (or niche activity) of corresponding mesenchymal cells. These organoid systems mimic various anatomical regions of the mammalian lung and are therefore dubbed tracheospheres, bronchiolospheres, bronchioalveolar organoids, and alveolospheres [3,25,26,27,28]. As with any culture assay, there is a strong need for standardization of the operating procedure to maximize data quality, robustness, and reproducibility.

One of the pioneering studies in the field is that of McQualter et al., who demonstrated the ability of lung EpCAM+ cells to form airway, alveolar, and mixed organoids when co-cultured with EpCAM− Sca-1+ mesenchymal cells in Matrigel [21]. The generation of organoids from murine and human AEC2s was later described, where the co-culture of AEC2s with PDGFRα+ mesenchymal cells or TASCs, a population that includes lipofibroblasts, led to the formation of alveolospheres containing AEC2s and AEC1s [10,20]. Alveolosphere cultures were also used to characterize the WNT-responsive fraction of AEC2s in the murine lung and investigate alveolar niche interactions. Such an AEC2 subset displayed superior alveolosphere formation capacity and responds to WNT and FGF stimulation [14,29,30]. Another AEC2 subset that acts as a quiescent repertoire for mature AEC2s is the IAAPs, which do not show robust alveolosphere formation capacity but are important for replenishing the bona fide AEC2 pool [15,16,31,32].

WNT and FGF signaling have been established as critical determinants of the AEC2 cell fate by independent studies [7,31,33,34,35,36]. The mesenchymal niche can be fractionated into AXIN2+ (a WNT-responsive population enriched with *Fgf7* expression) and FGF10+ subsets. Both fractions show lipofibroblast features including proximity to AEC2s and neutral lipid content [18,37]. How distinct these two subsets are, especially in terms of in vivo function during homeostasis, repair, and regeneration, remains to be seen. A third niche population is represented by WNT ligand-producing leucine-rich repeat-containing G protein-coupled receptor-5-positive (LGR5+) fibroblasts that have shown the ability to support alveolosphere formation [7]. In the current protocol, the bulk rMC population was used to achieve a simple and universal protocol to generate alveolospheres from WT mice without the need for transgenic animals or discriminating between rMC subsets. Table 3 highlights the main features of some of the published protocols to generate murine alveolospheres. The emerging picture is that (1) most of the studies listed below relied on the use of transgenic mice to generate alveolospheres, (2) a starting material of 5000 AEC2s combined with mesenchymal cells at a ratio of 1:10 and later mixed with Matrigel (1:1) is the most used approach, and (3) the data show varying CFE values even when using similar protocols.

## 5. Conclusions

Our protocol thus allows robust and reproducible generation of murine alveolsopheres using the EpCAM^low^ gating strategy, which represents a refinement of previous EpCAM gating strategies and avoids lumping EpCAM^high^ with EpCAM^low^ cells. As club cells and BASCs are not found in the EpCAM^low^ fraction [9,19], our approach minimizes contamination with bronchiolar and bronchioalveolar organoids, allowing to predominantly obtain alveolospheres. We believe that our protocol represents a step toward the standardization of this important research tool.

Finally, we were able to reproduce the effect of IL-1β on alveolosphere formation using our WT approach. The accumulation of KRT8+ transitional cells was evident based on whole-mount immunofluorescence data. The involvement of these and other transitional AEC2 populations in various respiratory diseases has gained tremendous interest in recent years [11,13] and further research is warranted to fully understand this phenomenon, decipher how to convert such dysplastic events into healthy scarless regeneration, and ultimately reconstitute the alveolar stem cell niche and restore optimal gas exchange in the diseased lung.

## Figures and Tables

**Table 1:** List of antibodies used for immunofluorescence.

Primary Antibody	Dilution	Secondary Antibody	Dilution
Rabbit α-Pro-SFTPC (Seven Hills Bioreagents, WRAB-9337, Cincinnati, OH, USA)	1:800	Donkey α-rabbit AF488 (Thermo Fisher Scientific, A-32790, Waltham, MA, USA)	1:1000
Mouse α-HOP (Santa Cruz, sc-398703, Santa Cruz, CA, USA)	1:150	Goat α-mouse AF647 (Abcam, ab150115, Cambridge, UK)	1:500
Rat α-KRT8 (Developmental Studies Hybridoma Bank (DSHB), TROMA-I-c, Iowa City, IA, USA)	1:200	Goat α-rat AF555 (Thermo Fisher Scientific, A-48263, Waltham, MA, USA)	1:1000

**Table 2:** Primer sequences used for qPCR.

Gene Name	Forward Primer	Reverse Primer
*Gapdh*	5′-CAT CAC TGC CAC CCA GAA GAC TG-3′	5′-ATG CCA GTG AGC TTC CCG TTC AG-3′
*Sftpc*	5′-GGT CCT GAT GGA GAG TCC AC-3′	5′-GAT GAG AAG GCG TTT GAG GT-3′
*Ager*	5′-GCC ACT GGA ATT GTC GAT GAG G-3′	5′-GCT GTG AGT TCA GAG GCA GGA T-3′

**Table 3:** Comparison between various murine alveolosphere protocols.

Study	Mouse Strain	Epithelial Cells	Mesenchymal Cells	Epi:Mes Ratio	Set Up	CFE	Research Question
[10]	*Sftpc^Cre-ERT2^*; *tdTomato^flox^* and *Pdgfra^H2B-GFP^*	5 × 10^3^ SFTPC+ cells	1 × 10^5^ PDGFRα^high^ cells (also shown to be LipidTOX+)	1:20	ALI in Matrigel (mixed 1:1) for 16–17 days	2.3% ± 0.3%	Self-renewal and differentiation of AEC2s
[30]	*Sftpc^Cre-ERT2^*; *EYFP^flox^* and *Axin2^Cre-ERT2^*; *tdTomato^flox^*	5 × 10^3^ EYFP+ or tdTom+ cells	5 × 10^4^ primary adult lung fibroblasts	1:10	ALI in Matrigel (mixed 1:1 with MTEC-SAGM) for 14 days	~1.7% with total AEC2s versus ~2.6% with AXIN2+ AEC2s before passaging	Comparison between bulk and WNT-responsive AEC2s
[7]	*Sftpc^Cre-ERT2^*; *tdTomato^flox^*, *Lgr5^Cre-ERT2^*; *tdTomato^flox^* and *Lgr6^EGFP-Cre-ERT2^*; *tdTomato^flox^*	0.5–1 × 10^4^ SFTPC+ cells	0.5–1 × 10^5^ LGR5+ or LGR6+ cells	1:10	ALI in Matrigel (mixed 1:1) for 14 days	~4.8% with LGR5+ cells versus ~2.5% with LGR6+ cells before passaging	Comparison between LGR5+ and LGR6+ cells in terms of supporting bronchial, alveolar, and bronchioalveolar organoids
[7]	*Scgb1a1^Cre-ERT^*; *YFP^flox^* and *Lgr5^Cre-ERT2^*; *EYFP^flox^* *	0.5–1 × 10^4^ SCGB1A1+ cells	0.5–1 × 10^5^ LGR5+ cells	1:10	ALI in Matrigel (mixed 1:1) for 14 days	~2.6% with most organoids being alveolospheres	Comparison between LGR5+ and LGR6+ cells in terms of supporting bronchial, alveolar, and bronchioalveolar organoids
[37]	*Sftpc^Cre-ERT2^*; *EYFP^flox^*, *Axin2^Cre-ERT2^*; *EYFP^flox^*, *Wnt2^Cre-ERT2^*; *EYFP^flox^* and *Pdgfra^H2B-GFP^*	5 × 10^3^ SFTPC+ cells	5 × 10^4^ cells	1:10	ALI in Matrigel (mixed 1:1) for 21 days	Between ~1.2% and ~6.2%	Comparison between the alveolosphere-supportive ability of various mesenchymal subsets
[20]	*Sftpc^Cre-ERT2^*; *tdTomato^flox^* and *Pdgfra^H2B-GFP^*	5 × 10^3^ SFTPC+ cells	5 × 10^4^ cells	1:10	ALI in Matrigel (mixed 1:1) for 14 days	Up to ~9%	Investigating BMP/SMAD signaling in AEC2 renewal and differentiation
[12]	*Sftpc^Cre-ERT2^*; *tdTomato^flox^*	5 × 10^3^ SFTPC+ cells	Cultured lung stromal cells	1:5	ALI in Matrigel (mixed 1:1) for 14 days	Between ~1.6% (without IL-1β) and ~3% (with IL-1β)	Investigate the effect of IL-1β stimulation on AEC2 self-renewal, transitional state, and differentiation
[16]	*Sftpc^Cre-ERT2^*; *tdTomato^flox^*	1 × 10^3^ SFTPC+ cells	2 × 10^4^ rMCs (EpCAM− CD45− CD31− Sca-1+) from C57BL/6 lungs	1:20	ALI in Matrigel (mixed 1:1) for 14 days	~1.2%	Assess the ability of AEC2 subsets to form alveolospheres
[38]	C57BL/6	5 × 10^3^ AEC2 isolated via enzymatic digestion (Dispase II), cell-specific antibody labeling (biotinylated Ter-119, CD104, CD16/32, CD45, CD31), and magnetic separation (Anti-Biotin MicroBeads)	1 × 10^5^ lung mesenchymal stromal cells (L-MSCs) isolated by differential adhesion	1:20	ALI in Matrigel (mixed 1:1) for 9–12 days	Not directly stated by can be estimated to be ~0.6%	Assess the effect of aging on alveolosphere formation
[33]	*Sftpc^Cre-ERT2^*; *tdTomato^flox^* and *Sftpc^Cre-ERT2^*; *Fgfr2b^flox/flox^*; *tdTomato^flox^*	5 × 10^3^ SFTPC+ cells	5 × 10^4^ primary adult lung fibroblasts	1:10	ALI in 1:1 mixture of MTEC-SAGM:Matrigel (mixed 1:1) for 14 days	Between ~9% and ~11%	Assessing the role of FGFR2b signaling on alveolosphere formation
[18]	*Sftpc^Cre-ERT2^*; *tdTomato^flox^*, *Fgf10-lacZ*, *ob/ob* and C57BL/6	5 × 10^3^ EpCAM+ Lysotracker+ tdTom+	5 × 10^4^ rMCs (EpCAM− CD45− CD31− Sca-1+) or EpCAM− CD45− CD31− Sca-1− cells; LipidTOX+ rMCs or LipidTOX− rMCs; FGF10+ rMCs/FGF10− rMCs; ob/ob rMCs from C57BL/6 lungs	1:10	ALI in Matrigel (mixed 1:1) for 14 days	Between ~2.2% and ~6.2%	Comparison of niche activity between various mesenchymal subsets
[39]	*Sftpc^Cre-ERT2^*; *tdTomato^flox^* and C57BL/6	2–5 × 10^3^ tdTom+ cells or Lysotracker+ EpCAM+ cells	5 × 10^4^ PDGFRα+ fibroblasts	1:10 to 1:25	Matrigel droplets (mixed 1:1) for 10–15 days	Between ~8% and 9% with tdTom+ cells	Assessing the impact of inflammation on alveolar regeneration
[40]	C57BL/6	2–3 × 10^3^ CD45− CD31− EpCAM+ Lysotacker+ cells	None	N/A	Matrigel droplets (mixed 1:1) for 10–12 days	Information not available	Protocol to generate feeder-free alveolospheres **

* Experiments were also conducted with LGR6+ cells but predominantly yielded bronchiolar organoids rather than alveolospheres. ** AEC1 differentiation was not achieved under these conditions.

## Data Availability

Raw data can be provided upon reasonable request.

## Supplementary Materials

The following supporting information can be downloaded at: https://www.mdpi.com/article/10.3390/cells13110922/s1, Table S1: RNA-seq on sorted WT EpCAM^low^ cells and tdTom+ cells, as well as the alveolospheres arising from them.
